# Preconception Lifestyle and Weight-Related Behaviors by Maternal Body Mass Index: A Cross-Sectional Study of Pregnant Women

**DOI:** 10.3390/nu11040759

**Published:** 2019-03-31

**Authors:** Adina Y. Lang, Cheryce L. Harrison, Jacqueline A. Boyle

**Affiliations:** Monash Centre for Health Research and Implementation, School of Public Health and Preventative Medicine, Monash University, Melbourne, VIC 3004, Australia; adina.lang@monash.edu (A.Y.L.); cheryce.harrison@monash.edu (C.L.H.)

**Keywords:** preconception, risk perception, health behaviors, body mass index, women’s health, pregnancy intention

## Abstract

Obesity is a rising global health challenge, particularly for reproductive-aged women. Our cross-sectional study of pregnant women (*n* = 223) examined associations between preconception body mass index (BMI) and socio-demographics, weight perceptions and lifestyle behaviors. Over half of women were overweight (33.2%) or obese (22.0%), 49.6% of which perceived their weight as normal. High proportions of women reported planning their pregnancies (70.0%) and were actively trying to lose or maintain their weight preconception (72.7%). Weight management approaches varied from reducing discretionary foods (63.7%) to professional support (8.1%). Obese women had significantly greater odds of reducing discretionary foods (odds ratio (OR) = 6.69 95% confidence interval (CI) 2.13–21.00, *p* = 0.001) and using structured diets (adjusted odds ratio (AOR) = 9.13 95% CI 2.90–28.81, *p* < 0.001) compared to normal-weight women. After adjusting for socio-demographics, compared to normal-weight women, overweight (AOR = 5.24 95% CI 2.19–12.56, *p* < 0.001) and obese (AOR = 2.85 95% CI 1.06–7.67, *p* = 0.04) women had significantly increased odds of exercising for weight management and significantly lower odds of taking folic-acid preconception (overweight: AOR = 0.40 95% CI 0.18–0.90, *p* = 0.01, obese: AOR = 0.38 95% CI 0.16–0.91, *p* = 0.03). Large proportions of women planning a pregnancy have an overweight/obese BMI, with associated suboptimal health behaviors and reduced health professional engagement preconception. Further research exploring women’s perspectives regarding preconception lifestyles is needed to inform effective preconception health promotion strategies.

## 1. Introduction

The health of women prior to conception (preconception) can affect their reproductive health, pregnancy outcomes and the health of their future child [1]. Improvements in preconception health and pregnancy outcomes can be achieved through the adoption of healthy behaviors including, but not limited to, maintaining a healthy diet and regular physical activity aligned with national guidelines [2,3], attaining an optimal weight (body mass index >18 to <25 kg/m^2^), cessation of smoking, illicit drug use and alcohol consumption, controlled management of chronic diseases, up-to-date health screenings and immunizations and vitamin supplementation [4].

Obesity is one of the largest global public health challenges [5], and young reproductive-aged women present the population group at highest risk, with progressive weight gain, rising obesity prevalence and related major reproductive, metabolic and psychological complications [6]. Women who enter pregnancy overweight or obese are at increased risk of a range of adverse reproductive, maternal and child health outcomes including gestational hypertension, gestational diabetes, pre-eclampsia, depressive disorders, cesarean section and the delivery of a large-for-gestational-age baby [1,5,7,8]. Over 50% of women in high-income countries enter pregnancy overweight or obese [9,10,11]. There appears to be limited awareness of the impact of weight and gestational weight gain on women’s health and that of their baby. Women also experience a complex array of social, emotional, physiological, environmental and behavioral factors that can affect their weight [12,13].

The preconception period is becoming established as a critical window in which to intervene for improved multigenerational health outcomes for women and their offspring [14]. While evidence gaps remain in understanding the barriers and enablers of optimal preconception health behaviors, it is increasingly recognized that creating supportive environments and promoting sustainable lifestyle and diet-related strategies for weight management are critical for optimal maternal and child health benefits [5,15,16,17]. To date, there are limited studies exploring the relationship between preconception BMI and lifestyle behaviors prior to pregnancy, and these have shown mixed results.

For example, McKenna and colleagues prospectively investigated preconception supplement use among Australian women, and reported 51% of women were taking folic-acid alone or as part of a multivitamin [18]. However, no relationship between BMI and overall preconception supplementation behavior was found [18]. This differs from previous reports showing reduced odds of folic-acid supplementation in obese women compared to those of a normal BMI [19,20]. Associations between pregnancy planning/intention (referred to herein as pregnancy planning) and BMI have also shown inconsistent findings, with some [21] but not all [22] studies reporting higher or lower odds of pregnancy planning with increasing BMI.

Improving the understanding of preconception health determinants will inform critically needed health promotion and targeted lifestyle interventions in this population [23,24,25]. Therefore, the current study examines (1) the socio-demographic characteristics of preconception women across BMI groups; (2) differences in women’s perceived weight and measured BMI; and (3) the relationship between weight-related behaviors and preconception BMI.

## 2. Materials and Methods 

### 2.1. Study Design 

A retrospective cross-sectional questionnaire completed by Australian women early in pregnancy at their first clinical antenatal appointment.

### 2.2. Recruitment, Setting and Participants 

Women were recruited through Monash Health maternity services, a large public maternity service in South East Melbourne, Australia. Monash Health is Victoria’s largest healthcare provider, delivering approximately 9000 births per year across a diverse population including 62% non-Australian born women [26]. 

Prior to the commencement of the study, researchers met with clinical midwifery and support staff to establish recruitment processes. A study invitation flyer was mailed to all women prior to the first clinical antenatal booking appointment throughout the study period (August 2017 to March 2018). Women could express interest in participating prior to their hospital booking or were recruited face-to-face at their first antenatal visit by a researcher. Eligible participants included pregnant women aged over 18 years that were able to speak and write in English. Women were excluded if they were less than 18 years of age and unable to communicate or comprehend English sufficiently. 

Women were provided with a secure link to complete the questionnaire online or alternatively were provided a paper copy. Follow-up reminders were sent if required. In recognition of women’s time, and as gratitude for taking part, all participating women were placed in the draw for a $100AUD gift card. Study ethics approval was obtained from the Monash Health (RES-17-0000-690XL) and Monash University (Project no. 10370) Human Research Ethics Committees. All women gave informed consent prior to participating in the study. 

### 2.3. The Questionnaire 

The questionnaire developed was based on a review of existing tools and guidelines in the literature [27,28,29,30,31,32,33,34]. The aim of the questionnaire was to assess women’s pregnancy planning and preconception lifestyle behaviors, awareness and information needs. It was then refined and pre-tested through cognitive interviews [35] for consumer comprehension and acceptability. 

Socio-demographics (listed in Table 1) collected within the questionnaire were supplemented by information from medical records including measured weight (kg) and height (cm), gestation at first visit, gravida, parity, previous birth outcomes (live birth, miscarriage, stillbirth or termination), family history of hypertension and diabetes and postcode. Socio-economic status (SES) was estimated by aligning postcodes to corresponding deciles in the Australian Socio-Economic Indexes for Areas (SEIFA), Index of Relative Socio-economic Disadvantage [36]. Deciles 1–5 are classified as higher-level disadvantage and decile 6–10 as lower-level disadvantage. Body mass index was calculated (weight/height (m^2^)) and classified according to the World Health Organization definitions [37]: underweight (≤18.50); normal-weight (18.50–24.99 kg/m^2^); overweight (25.00–29.99 kg/m^2^); and obese (≥30.00 kg/m^2^). Self-reported weight categories were also obtained using the following question: “In the 3 months before you became pregnant, were you a healthy (normal) weight, overweight, underweight or unsure”. Women were asked to select one response option.

Women were classified as having a relevant medical condition or taking relevant teratogenic medications if they reported having any medical conditions or taking any medications identified in the literature as having potential adverse effects on pregnancy and for which medical review would be advised in the 6 months before pregnancy [27]. For example, medical conditions included asthma, anxiety, epilepsy, depression, and thyroid disease.

The London Measure of Unplanned Pregnancy (LMUP) [28], adapted and validated for use in the Australian population [38], was used to assess pregnancy planning. Six questions related to the use of contraceptives, pregnancy timing, intentions, desire, partner influences and preparations were completed with each item scored zero, one or two and summed to produce an overall pregnancy planning score ranging from zero to 12. A higher score represents a greater level of pregnancy planning. It is recommended that the full range of scores are used for analysis, and for prevalence estimates, scores can be classified into 0–3, unplanned; 4–9, ambivalent; 10–12, planned [39]. However, due to the highly skewed dataset, an accepted binary score for the LMUP was used, with scores ≥10 classified as being planned and 0–9 unplanned.

Preconception folic-acid supplementation taken alone or as part of a multivitamin was collected as part of the LMUP, with responses coded as “Yes” and “No”. To determine weight loss/maintenance intention, women were asked whether they had attempted to maintain their weight or lose weight in the 6 months preconception, with response options including “Yes, maintain a healthy weight”, “Yes, lose weight” or “No”. The first two options were combined for analysis to assess weight loss/maintenance overall. Women that responded “Yes” to the intention of losing or maintaining a healthy weight were asked what approaches they had used and could select one or more relevant options. Response options were collapsed and re-coded into four main approach types for analysis, including cutting down on takeaway foods, fats, sugar or meal size (reducing discretionary foods/meal sizes); commencing a structured diet/meal replacements/weight loss programs (structured diet); weight loss program in consultation with a health professional, e.g., doctor, dietician, nurse, pharmacist (professional support); and increased vigorous exercise, e.g., running, cycling, vigorous sports/single or group sessions with a personal training (vigorous exercise). Smoking, alcohol intake and illicit drug use was also recorded for the 3 months prior to conception. 

### 2.4. Statistical Analysis 

Data analysis was performed using SPSS version 25 (Armonk, New York, NY, USA). Descriptive statistics were presented as median (interquartile range (IQR: 25th–75th percentile)) for continuous variables, and frequencies and percentages for categorical variables. The Kruskal–Wallis Test or the chi-squared test (χ^2^ tests) were used to compare the socio-demographic characteristics of women stratified by BMI classification; to assess any differences between self-reported weight category and measured BMI; and to examine weight maintenance or weight loss behaviors by BMI. Univariable and multivariable logistic regression were used to evaluate associations between BMI and preconception health behaviors. The multivariable models included covariates that generated a *p*-value < 0.1 on univariable logistic regression or on clinically significant a priori (see covariates listed in Table 2). *p*-values < 0.05 were considered statistically significant.

## 3. Results

### 3.1. Demographic Characteristics and BMI

Overall, 316 eligible women expressed interest in participating in the study either face-to-face (*n* = 305), or by telephone/email (*n* = 11). Of these, 91 women did not complete the questionnaire (including *n* = 4 who formally withdrew), with data available for 225 completed questionnaires (71% completion rate). A further two participants were excluded because their BMI was classified as underweight and there were insufficient numbers for analysis, leading to *n* = 223 participants being included in the analysis. 

Table 1 presents women’s demographic characteristics stratified by BMI. Women’s median (IQR) age, gestation and BMI at recruitment was 30 (27.0, 33.0) years, 7 (5.0, 11.0) weeks gestation and 25.4 (23.0, 29.5) kg/m^2^, respectively. Forty-five percent of the cohort were classified as being of a normal BMI, with 33.2% overweight and 22.0% obese. The majority of women were married/de facto, held a post-secondary school education and were in paid employment one year prior to pregnancy. Just over half of women were Australian-born and had a previous live birth. 

Significant differences were found between BMI groups for previous pregnancy loss (*p* = 0.02), pre-existing medical conditions (e.g., anxiety, depression, asthma, thyroid disease, polycystic ovary syndrome, *p* < 0.001) and medication use before conception (*p* < 0.001), with obese women reporting the highest proportions. No significant differences were observed between BMI category and place of birth (Table 1).

### 3.2. Weight Perception 

Perceived weight was recorded for *n* = 220 women. Nearly a third of women overall (31%) incorrectly identified themselves as normal-weight, underweight or overweight and 9% were not sure. This was most marked in overweight women, with 70.8% (*n* = 51, *p* < 0.001) incorrectly identifying themselves as a normal-weight. Among obese women, 18.4% (*n* = 9) misperceived themselves to be a normal-weight. Nine percent (*n* = 9) of normal-weight women perceived themselves to be underweight or overweight. Women who perceived themselves to be overweight were significantly more likely to report trying to lose weight compared to women who perceived themselves to be a normal-weight (64.7% vs. 13.3%, *p* < 0.001).

### 3.3. Preconception Health Behaviors Overall and by BMI 

Table 2 presents the frequencies as well as unadjusted (OR) and adjusted (AOR) associations between preconception lifestyle behaviors across BMI groups. 

Overall, 70.0% of women were classified as having a planned pregnancy and 52.9% reported taking folic-acid preconception. Of those women who had ever smoked or drank alcohol, 44.3% reported continuing smoking and 55.9% reported drinking at least one alcoholic beverage in the 3 months prior to conception. Fifteen percent of women reported having ever used illicit drugs, of which 9.4% (*n* = 3) women reported using illicit drugs in the 3 months prior to conception. No significant differences were found between BMI category and pregnancy planning, smoking, drinking alcohol and illicit drug use (Table 2). On univariable analysis there was no difference in folic-acid use between BMI groups. However, multivariable analysis adjusting for age, place of birth, number of children and pregnancy planning showed overweight and obese women had significantly lower odds of taking folic-acid preconception compared to women of a normal-weight (overweight: AOR = 0.40 95% CI 0.18–0.90; obese: AOR = 0.38 95% CI 0.16–0.91, *p* = 0.03) (Table 2).

Overall, 72.7% (*n* = 160) of all women in the cohort had tried to manage (lose or maintain) their weight preconception and no significant differences were found by BMI (Table 2). Of these 160 women, the majority 63.7% (*n* = 102) reported trying to maintain a normal-weight and 36.3% (*n* = 58) were trying to lose weight. A significantly greater proportion of obese women were trying to lose weight compared to overweight and normal-weight women (76.5% vs. 37.5% vs. 15.7%, *p* < 0.001). 

Women could report taking more than one weight maintenance approach (reducing discretionary foods/meal sizes, structured diet, professional support, vigorous exercise). The most common weight management approaches reported was reducing discretionary foods and/or meal sizes (63.7%), while consulting a health professional was least common (8.1%). Overall, there was no significant difference in the proportion of women attempting weight management in relation to place of birth. Differences were apparent, however, in the uptake of vigorous exercise as a weight management strategy, with Australian-born women having greater odds of using this approach compared to non-Australian-born women. 

Obese women were more likely to attempt to reduce discretionary foods/meal sizes (OR = 6.69 95% CI 2.13–21.00, *p* = 0.001) and adopt a structured diet compared to normal-weight women (OR = 8.00 95% CI 2.85–22.43, *p* < 0.001 and AOR = 9.13 95% CI 2.90–28.81 *p* < 0.001) (Table 2). On adjustment, both overweight and obese women were significantly more likely to engage in vigorous exercise for weight management compared to women with a normal BMI (overweight: OR = 4.19 95% CI 1.86–9.45, *p* = 0.001, AOR = 5.24 95% CI 2.19–12.56, *p* < 0.001; obese: OR = 2.64 95% CI 1.03–6.74, *p* = 0.04; AOR = 2.85 95% CI 1.06–7.67, *p* = 0.04). 

Of the 160 women actively managing their weight, 122 reported using ≥1 of the healthy weight maintenance approaches. Of the 122, 59.0% (*n* = 72) took on one approach type, 24.6% (*n* = 30) took two approaches and 16.4% (*n* = 20) took a combination of 3 or more approach types. The remaining responses (*n* = 38) indicated doing “none of the above” or were blank.

## 4. Discussion

Our study provides new insight into the relationship between women’s preconception BMI and pregnancy preparation in relation to lifestyle-related preconception health behaviors in an Australian context. We report over half of our cohort of pregnant women had a preconception BMI in the overweight or obese range, with many experiencing medical and pregnancy health risks. Almost half of women incorrectly identified their weight as normal when according to their BMI they were overweight or obese. Many women were trying to lose or maintain their weight, yet few sought advice from medical, nursing or allied health professionals. In addition, there appeared to be a lack of awareness and uptake of folic-acid preconception and continued risk-taking behaviors (e.g., continued smoking and alcohol consumption) preconception.

A high proportion of women were overweight (33.2%) or obese (22.0%), corresponding with previous Australian and international population estimates [9,10,11]. Women with a BMI classified as overweight or obese were as likely as normal-weight women to plan for their pregnancies, confirming prior Australian findings [22]. However, most overweight women perceived themselves to be of a normal-weight compared to other BMI groups [40,41]. This suggests a lack of risk perception in women with excess weight, but who are not yet obese, and may reflect the normalization of increasing weight at a population level [40]. 

Encouragingly, despite these misperceptions, irrespective of their BMI, almost three-quarters of women in this study were actively trying to manage (lose or maintain) their weight in the preconception period. Consistent with prior literature [42], women who perceived themselves to be overweight were significantly more likely to report attempting to lose weight preconception. In addition, a larger proportion of women (76.5%) with a measured BMI in the obese category were trying to lose weight. This shows promise for women’s consciousness and receptiveness to preconception weight management support before pregnancy. Weight management is complex and challenging with multifactorial contributing factors including environmental, social, genetic, physiological and behavioral influences [43]. Weight gain is difficult to manage and extremely challenging to reverse [43]. With progressive weight gain (approximately 600 g/year) and acceleration towards obesity development prevalent in young reproductive-aged women [6], increasing awareness of the importance of weight gain prevention, healthy lifestyles and effective strategies to support women are now critically required. 

Women were more likely to report the self-management of their weight preconception compared with engaging in health professional support, consistent with prior literature [44,45]. This is somewhat surprising, however, given that 55% of women in this study were overweight or obese and were more likely to report a pre-existing medical condition and/or a previous pregnancy loss compared with normal-weight women. Health professional engagement has been suggested to improve the management of pre-existing health conditions preconception, optimized gestational weight gain in early pregnancy and improved compliance with folic-acid supplementation [46]. Yet preconception women are a diverse population who may not identify themselves as a distinct high-risk group for weight gain, and with limited healthcare engagement, may miss opportunities for targeted health promotion [47]. Additional barriers include varying levels of motivation, perceived costs, competing family commitments and varying health professional knowledge and skills to implement preconception weight management strategies [40,48]. Our findings highlight the need to reach women in varied ways within and beyond maternity, child and general healthcare settings, potentially including education, workplace and recreational settings. The development and testing of free or low-cost evidence-based programs promoted to the public and supported by health professionals could address this gap.

Furthermore, women attempting to manage their weight were more likely to adopt just one approach rather than a combination of strategies, despite relatively high pregnancy planning. This is contradictory to evidence-based weight gain prevention approaches, which require a combination of a balanced diet, adequate physical activity and behavior change support in order to maintain weight [43]. Given preconception women are more receptive towards optimizing healthy lifestyle behaviors to ensure the health of their baby, these results highlight the need for timely, evidence-based population level strategies that empower and equip women with knowledge, skills and self-management strategies for healthy lifestyles and weight gain prevention relevant across their lifespan [24]. 

Despite planning for pregnancy, only approximately half of women in this study were supplementing with folic-acid preconception, consistent with previous Australian studies [18]. This level is still suboptimal and suggests a lack of awareness of the importance and risks related to folic-acid use, potentially due to limited health professional engagement or a preconception health check prior to their antenatal appointment. Interestingly, on adjustment for socio-demographic factors and pregnancy planning, overweight and obese women had a significantly lower odds of supplementing with folic-acid compared to normal-weight women, consistent with some [19,20] but not all [18] previous research. Irrespective, this remains of particular concern considering that preconception folic-acid supplementation is vital to reduce the risk of neural tube defects, for which overweight or obese women are at a higher risk [7,20]. 

Overall, women also reported risk-taking behavior, with around half of women who had ever drank alcohol or smoked reporting continuing to drink and smoke in the 3 months before pregnancy. Australian primary care guidelines include recommendations for preconception care to be provided to all women of reproductive age [32] which includes lifestyle modifications such as folic-acid supplementation, weight management, smoking and alcohol cessation. However, to date, the routine implementation of preconception recommendations into primary care visits has been limited [49]. In light of these findings, primary healthcare providers should be encouraged to include these sensitive yet vital discussions as part of their routine family planning consultations. These findings further emphasize that overweight and obese women who often experience multiple risk factors and health complications, e.g., gestational diabetes, type 2 diabetes, and cardiovascular disease, require particular attention.

Lifestyle change is complex and difficult to understand and achieve. Future prospective research is critical to deepen our understanding of the determinants of weight management behaviors preconception [50] to enable the provision of appropriate health promotion policy, strategies and interventions. An integrated approach that moves beyond the individually focused medical model is needed. This means taking a holistic population-based focus at critical times across the reproductive life course, creating supportive environments and addressing the socio-ecological determinants of overweight and obesity in preconception, pregnancy and postpartum. A balance is needed to sensitively raise awareness of the importance of healthy lifestyles, weight and overall wellbeing preconception. Since women are not presenting to health professionals for weight management and instead are using other methods, intervention in multiple settings and across different levels are required. These may range from individual engagement with primary healthcare to reaching families in schools and workplaces as well as broader communities, and wider social and cultural environments, supported by governments, health systems and industry policy [15].

### Limitations and Strengths 

This study addresses the limited knowledge base regarding the relationships between women’s preconception BMI and their lifestyle and weight-related preconception health behaviors in Australia. A high proportion of women from culturally and linguistically diverse backgrounds were included in the study. However, only English-speaking participants were included. Although the majority of women in this study resided in areas of lower levels of socio-economic disadvantage, there was a 30% representation from women living in areas with higher levels of disadvantage, as defined by the SEIFA criteria [36]. Limitations include the study being implemented retrospectively in an antenatal population rather than prospectively prior to pregnancy. This study was limited in its ability to assess the effectiveness of weight management strategies used by women for weight loss/maintenance, and this should be considered in future research. Cross-sectional analysis can explore associations; however, causal relationships cannot be confirmed. Inferential statistics were not possible or meaningful in some cases due to low frequencies in some variables of interest. 

## 5. Conclusions

Our results confirm a high proportion of women continue to enter pregnancy overweight or obese and experience additional health complications which could increase the risk of adverse maternal and neonatal health outcomes. Professional healthcare engagement and the uptake of multi-strategic approaches to weight management were low. In addition, crucial preconception health behaviors were suboptimal, including folic-acid supplementation, particularly in overweight and obese women. Awareness-raising and support is needed through holistic multi-strategic approaches including integration and capacity building for preconception health promotion in primary care supported by broader policy and population-level programs for women preconception. Further research is needed to inform the development of weight management strategies preconception, including the assessment of the effectiveness of different identified approaches and a qualitative exploration of the barriers women may identify in achieving their optimal weight. 

## Figures and Tables

**Table 1:** Participant characteristics stratified by BMI.

Characteristic		BMI			
	Overall (*n* = 223)	Normal-Weight (*n* = 100)	Overweight (*n* = 74)	Obese (*n* = 49)	*p*-Value
**Age (years) Median (IQR)**					
	30.0 (27.0, 33.0)	30.0 (27.0, 33.0)	30.0 (27.0, 33.0)	30.0 (26.5, 34.0)	0.75
**Gestation (weeks) Median (IQR)**					
	7.0 (5.0, 11.0)	7.0 (5.0, 11.0)	7.0 (6.0, 11.3)	7.0 (5.5, 8.0)	0.78
	***n* (%)**				
**Age group**	*n* = 223				
*<25*	32 (14.3)	17 (17.0)	9 (12.2)	6 (12.2)	0.59
*25* *–29*	70 (31.4)	28 (28.0)	27 (36.5)	15 (30.6)	
*30* *–34*	84 (37.7)	42 (42.0)	25 (33.8)	17 (34.7)	
*>35*	37 (16.6)	13 (13.0)	13 (17.6)	11 (22.4)	
**Relationship status**	*n* = 211				
*Married/De facto*	194 (91.9)	83 (90.2)	64 (90.1)	47 (97.9)	0.22
Unmarried *	17 (8.1)	9 (9.8)	7 (9.9)	1 (2.1)	
**Place of birth**	*n* = 211				
*Australia*	119 (56.4)	49 (53.3)	39 (54.9)	31 (64.6)	0.42
*Outside Australia*	92 (43.6)	43 (46.7)	32 (45.1)	17 (35.4)	
**Previous live birth**	*n* = 223				
*Yes*	127 (57.0)	52 (52.0)	44 (59.5)	31 (63.3)	0.37
*No*	96 (43.0)	48 (48.0)	30 (40.5)	18 (36.7)	
Previous pregnancy loss **	*n* = 223				
*Yes*	69 (30.9)	24 (24.0)	22 (29.7)	23 (46.9)	0.02
*No*	154 (69.1)	76 (76.0)	52 (70.3)	26 (53.1)	
**Number of children**	*n* = 223				
*0* *–2*	209 (93.7)	95 (95.0)	69 (93.2)	45 (91.8)	0.74
*>3*	14 (6.3)	5 (5.0)	5 (6.8)	4 (8.2)	
**Education**	*n* = 211				
*Post-secondary school*	167 (79.1)	77 (83.7)	55 (77.5)	35 (72.9)	0.30
*School only*	44 (20.9)	15 (16.3)	16 (22.5)	13 (27.1)	
**Employment**	*n* = 211				
*Paid employment*	156 (73.9)	68 (73.9)	56 (78.9)	32 (66.7)	0.33
*Unpaid employment/unemployed*	55 (26.1)	24 (26.1)	15 (21.1)	16 (33.3)	
SIEFA ***	*n* = 223				
*Higher level disadvantage (Decile 1* *–5)*	68 (30.5)	30 (30.0)	20 (27.0)	18 (36.7)	0.51
*Lower level disadvantage (Decile 6* *–10)*	155 (69.5)	70 (70.0)	54 (73.0)	31 (63.3)	
**Relevant medical condition**	*n* = 207				
*Yes*	97 (46.9)	34 (37.8)	27 (38.6)	36 (76.6)	<0.001
*No*	110 (53.1)	56 (62.2)	43 (61.4)	11(23.4)	
**Relevant medications**	*n* = 223				
*Yes*	33 (14.8)	10 (10.0)	7 (9.5)	16 (32.7)	<0.001
*No*	190 (85.2)	90 (90.0)	67 (90.5)	33 (67.3)	
**Family history hypertension**	*n* = 130				
*Yes*	96 (73.8)	37 (66.1)	34 (73.9)	25 (89.3)	0.07
*No*	34 (26.2)	19 (33.9)	12 (26.1)	3 (10.7)	
**Family history diabetes**	*n* = 136				
*Yes*	102 (75.0)	41 (68.3)	36 (75.0)	25 (89.3)	0.11
*No*	34 (25.0)	19 (31.7)	12 (25.0)	3 (10.7)	

Data are presented as median (interquartile range (IQR: 25th–75th percentile)), or as *n* (%). * Never married/widow/divorced/separated. ** Previous miscarriage/stillbirth/termination. *** Socio-economic status was estimated according to participant’s postal code, by the deciles in the Australian Socio-Economic Indexes for Areas (SEIFA), Index of Relative Socio-economic Disadvantage [36] Deciles 1–5 were classified as higher-level disadvantage and decile 6–10 as lower-level disadvantage. Total *n* for each variable may vary based on the total number of responses.

**Table 2:** Preconception health behaviors and BMI.

	All	Univariable OR (95% CI)	*p*-Value	Multivariable OR (95% CI)	*p*-Value
	*n* (%)				
**Planned pregnancy ^1^ (*n* = 223)**	156 (70.0)				
*Normal-weight*	69 (69.0)	1 (ref)			
*Overweight*	46 (62.2)	0.74 (0.39–1.39)	0.35	0.672 (0.34–1.34)	0.26
*Obese*	41 (83.7)	2.30 (0.97–5.49)	0.06	1.93 (0.77–4.83)	0.16
**Folic-acid use ^2^ (*n* = 223)**	118 (52.9)				
*Normal-weight*	59 (59.0)	1 (ref)			
*Overweight*	33 (44.6)	0.56 (0.31–1.03)	0.06	0.40 (0.18–0.90)	0.03
*Obese*	26 (53.1)	0.79 (0.40–1.56)	0.49	0.38 (0.16–0.91)	0.03
**Weight management (*n* = 220)**	160 (72.7)				
*Normal-weight*	70 (70.7)	1 (ref)			
*Overweight*	56 (77.8)	1.45 (0.72–2.93)	0.30		
*Obese*	34 (69.4)	0.94 (0.45–1.98)	0.87		
***Reducing discretionary foods/meal sizes* (*n* = 160)**	102 (63.7)				
*Normal-weight*	37 (52.9)	1 (ref)			
*Overweight*	35 (62.5)	1.49 (0.73–3.04)	0.28		
*Obese*	30 (88.2)	6.69 (2.13–21.00)	0.001		
***Structured diet*^3^** **(*n* = 160)**	32 (20.0)				
*Normal-weight*	7 (10.0)	1 (ref)			
*Overweight*	9 (16.1)	1.72 (0.60–4.96)	0.31	2.00 (0.66–6.02)	0.22
*Obese*	16 (47.1)	8.00 (2.85–22.43)	<0.001	9.13 (2.90–28.81)	<0.001
***Professional support* (*n* = 160)**	13 (8.1)				
*Normal-weight*	2 (2.9)	1 (ref)			
*Overweight*	3 (5.4)	1.93 (0.31–11.94)	0.48		
*Obese*	8 (23.5)	10.46 (2.08–52.55)	0.004		
***Vigorous exercise*^4^** **(*n* = 160)**	50 (31.3)				
*Normal-weight*	12 (17.1)	1 (ref)			
*Overweight*	26 (46.4)	4.19 (1.86–9.45)	0.001	5.24 (2.19–12.56)	<0.001
*Obese*	12 (35.3)	2.64 (1.03–6.74)	0.04	2.85 (1.06–7.67)	0.04
Smoking * (*n* = 61)	27 (44.3)				
*Normal-weight*	14 (53.8)	1 (ref)			
*Overweight*	8 (47.1)	0.76 (0.22–2.60)	0.66		
*Obese*	5 (27.8)	0.33 (0.09–1.20)	0.09		
Drinking alcohol ** **(*n* = 143)**	80 (55.9)				
*Normal-weight*	37 (60.7)	1 (ref)			
*Overweight*	28 (54.9)	0.79 (0.37–1.68)	0.54		
*Obese*	15 (48.4)	0.61 (0.25–1.45)	0.26		
Illicit drug use *** **(*n* = 216)**	32 (14.8)				
*Normal-weight*	13 (13.7)	1 (ref)			
*Overweight*	10 (13.9)	1.02 (0.42–2.47)	0.97		
*Obese*	9 (18.4)	1.42 (0.56–3.60)	0.46		

Data are presented as *n* (%) or as odds ratios (OR) and 95% confidence intervals (CI). * Only includes those who had ever smoked (*n* = 61). Frequencies and percentages of women who were smoking 3 months preconception are reported. ** Only includes those who ever drank alcohol (*n* = 143). Frequencies and percentage of women who were drinking ≥1 alcoholic drink 3 months preconception are reported. *** Reported based in the numbers of women who ever used illicit drugs out of the whole cohort for which data is available (*n* = 216). ^1^ Adjusting for relationship status and number of children.^2^ Adjusting for age (<25 or >25), place of birth, number of children and pregnancy planning (planned or unplanned). ^3^ Adjusting for having a relevant medical condition. ^4^ Adjusting for place of birth. Total *n* for each variable may vary based on the total number of responses.
